# Direct oral amoxicillin challenge in a tertiary care center: validating PEN-FAST in inpatient and outpatient populations with low-risk penicillin allergies

**DOI:** 10.1017/ash.2025.10168

**Published:** 2025-12-09

**Authors:** Natalie Harris, Nicholas A. Turner, Amy Mackowiak, Rebekah H. Wrenn

**Affiliations:** 1 Department of Pharmacy, Duke University Hospital, Durham, NC, USA; 2 Division of Infectious Diseases, Duke University School of Medicine, Durham, NC, USA

## Abstract

Ten percent of hospitalized patients have documented penicillin allergies, over 90% of which are mislabeled and lead to the use of less-preferred alternative antibiotics. The results of this single-center review provide an outlook at successes and opportunities for improvement with the use of an amoxicillin oral challenge for allergy de-labeling.

## Introduction

Penicillin allergy labels are common and lead to the use of less-preferred antibiotics, despite often being inaccurate.^1^ Use of alternative antibiotics may be associated with lesser efficacy and a higher risk of adverse outcomes, including toxicity, *Clostridium difficile* infection, and resistance.^1^ Pharmacist-led allergy assessments prompting de-labeling effectively reduce high-risk alternative antibiotic use.^2^

Direct oral amoxicillin challenge in patients with low-risk penicillin allergies is safe and effective for removing penicillin allergy labels, while reducing time and resource allocation associated with penicillin skin testing.^3^ Duke University Hospital piloted an amoxicillin oral challenge protocol beginning August 2023 for patients with low-risk penicillin allergies in inpatient and outpatient settings. We evaluated outcomes of a direct oral challenge (DOC) with amoxicillin, including success rate, allergy de-labeling, and adverse events.

## Methods

This retrospective, single-center evaluation included patients who received a one-time 250 mg oral amoxicillin challenge at Duke University Hospital from August 2023 to September 2024. Our institutional guideline recommends testing clinically stable patients with PEN-FAST score < 2 (representative of most subject’s scores enrolled in PALACE^3^) and a 96-hour H1 antagonist washout. The decision to proceed with DOC was made at the discretion of the treating physician. Patients were excluded if they were < 18 years of age, admitted to labor and delivery units, or challenged in the outpatient allergy clinic. Eligible patients were identified via electronic health record (EHR) query.

Data collected from EHR included demographics, comorbidities, allergy history, challenge tolerance, allergy de-labeling documentation, and adverse reactions. Safety was assessed by immediate tolerance and delayed adverse effects. Efficacy was assessed through reviewing allergy de-labeling and subsequent antibiotic use. Additional outcomes included beta-lactam use post-DOC and review of patients who were re-labeled or who were not de-labeled.

Data were analyzed using descriptive statistics.

## Results

A total of 99 patients received DOC from August 2023 to September 2024, and 94 patients were included for further review. Five patients were excluded based on primary location or team: obstetrics (*n* = 2), pediatrics (*n* = 2), or allergy (*n* = 1). The median age was 63 years (IQR 47.25–71), and 53.2% (*n* = 50) were male. Eighty four percent of patients (*n* = 79) received DOC inpatient. Approximately 22% (*n* = 21) of patients had iatrogenic immunosuppression, and the most common was chemotherapy for active cancer in 9.6% (*n* = 9). Common allergic reactions were childhood exanthem (39.4%), unknown (22.3%), and urticaria (21.3%). Documented allergy occurred > 10 years ago in 88.3% (*n* = 83) of patients. Most patients had PEN-FAST scores of 0 (48.9%) or 1 (45.7%). Table 1 summarizes baseline demographics and allergy history.

**Table 1.** Baseline demographics and allergy history

Characteristic	Patients (*n* = 94)
Age, median (IQR), y	63 (47.25–71)
Sex, n (%), male	50 (53.2%)
CCI, median (IQR)	2 (0–4)
Transplant recipient, *n* (%)	
Solid organ	6 (6.4)
None	88 (93.6)
Iatrogenic Immunosuppression, *n* (%)	
Prednisolone > 0.5 mg/kg/d for > 14 days	1 (1.1)
Cyclosporine	1 (1.1)
Tacrolimus or sirolimus	4 (4.3)
Azathioprine or mycophenolate	8 (8.5)
Leflunomide or methotrexate	2 (2.1)
Monoclonal antibodies	3 (3.2)
Cancer chemotherapy	9 (9.6)
None	73 (77.7)
Primary antibiotic name for allergy, n (%)	
Penicillin	79 (84.0)
Amoxicillin	12 (12.8)
Other/unknown	3 (3.2)
Documented allergic reaction, n (%)	
Childhood exanthem (unspecified)	37 (39.4)
Unknown	21 (22.3)
Urticaria	20 (21.3)
Diffuse rash or localized swelling	10 (10.16)
Immediate diffuse rash	6 (6.4)
Anaphylaxis or unexplained collapse	3 (3.2)
Family history of antibiotic allergy	3 (3.2)
Respiratory compromise	2 (2.1)
Generalized swelling	2 (2.1)
Mild neurologic manifestation	2 (2.1)
Fever	1 (1.1)
Gastrointestinal symptoms	1 (1.1)
Last occurrence, n (%)	
> 10 years	83 (88.3)
6–10 years	2 (2.1)
1–5 years	2 (2.1)
Unknown	7 (7.4)
Time to symptom onset, n (%)	
> 24 hours	3 (3.2)
< 2 hours	8 (8.5)
Unknown	83 (88.3)
Hospitalization, n (%)	
No	53 (56.4)
Yes	4 (4.3)
Unknown	37 (39.7)
Treatment required, n (%)	
Antihistamines	5 (5.3)
Other/unknown	49 (52.1)
None	40 (42.6)
PEN-FAST score, n (%)	
0	46 (48.9)
1	43 (45.7)
2	5 (5.3)
Allergy phenotype, n (%)	
Unlikely immune/significant	66 (70.2)
Immediate non-severe	10 (10.6)
Immediate severe	1 (1.1)
Delayed non-severe	3 (3.2)
Unknown	14 (14.9)
Assessor role, n (%)	
Infectious diseases physician/fellow	50 (53.2)
Internal medicine/hospitalist/other specialty	21 (22.3)
Pharmacist	22 (23.4)
Other	1 (1.1)
Challenge setting, n (%)	
Inpatient	79 (84.0)
Outpatient	15 (16.0)
Challenge location, n (%)	
Ward	76 (96.2)
Emergency department	2 (2.5)
Intensive care unit	1 (1.3)
Primary indication for challenge, n (%)	
Active infection	75 (79.8)
Surveillance (no acute need identified)	16 (17.0)
High antibiotic usage (2+ times/year)	3 (3.2)

Of 94 patients who underwent DOC, 93 (98.9%) tested negative, and 81 (86.2%) subsequently had their allergy removed from their medical record. Of the 12 patients with a negative test who were not de-labeled, 9/12 (75%) had no documented indication for allergy label retention, 2/12 (16.7%) had an index reaction to cephalosporins, and 1/12 (8.3%) experienced a delayed reaction. Nine (9.6%) patients were re-labeled within one year of DOC, with 7/9 (77.8%) lacking clear reason for re-labeling and 2/9 (22.2%) experienced a delayed reaction.

One patient (1.1%) exhibited an immediate reaction, and four (4.3%) exhibited delayed reactions. Delayed reactions included diffuse rash (*n* = 3) and swelling (*n* = 1), one requiring treatment with an antihistamine. Of the four patients who experienced delayed reactions, three patients had a PEN-FAST score of 1 and one patient had a PEN-FAST score of 2. The median time to delayed reaction was 7 days. All immediate and delayed reactions were non-severe and self-limiting, resolving after discontinuation of penicillin derivative where appropriate. Table 2 provides a summary of patients who experienced reactions.

**Table 2.** Patients with immediate or delayed reactions

Patient	PEN-FAST characteristics	Reaction description	Outcome
1	Index reaction PEN-FAST: 0 Reaction > 10 years ago Reaction: Urticaria Time to onset: Unknown No hospitalization No treatment required	Immediate reaction Developed focal mild urticaria 35 minutes after oral amoxicillin challenge	Allergy was not removed from patient chart
2	Index reaction PEN-FAST: 1 Reaction > 10 years ago Reaction: Urticaria Time to onset: Unknown Unknown hospitalization Unknown treatment required	Delayed reaction Developed facial swelling 2 days after oral challenge Unclear if directly related to amoxicillin as patient called and reported similar reaction with alcohol in the past	Allergy added back to patient chart after reaction
3	Index reaction PEN-FAST: 1 Reaction > 10 years ago Reaction: Urticaria Time to onset: 1–2 days No hospitalization Antihistamine required	Delayed reaction Developed diffuse rash 10 days after challenge, while on amoxicillin-clavulanate therapy No other trigger identified, some documentation states “dry skin”	Allergy added back to patient chart days after reaction
4	Index reaction PEN-FAST: 1 Reaction > 10 years ago Reaction: Urticaria Time to onset: Unknown Unknown hospitalization Unknown treatment required	Delayed reaction Developed rash 38 days after challenge while on suppression therapy with amoxicillin Unclear cause of rash, switched to alternative agent	Allergy was not added back to patient chart after reaction
5	Index reaction PEN-FAST: 2 Reaction 1–5 years ago Reaction: Itching/rash Time to onset: 3 days No hospitalization Unknown treatment required	Delayed reaction On day 5 of amoxicillin-clavulanate therapy developed rash on trunk, back, and limbs Notably after receiving contrast dye for imaging which patient had a historic allergy to	Allergy added back to patient chart after reaction

Forty-nine (52.1%) patients received a penicillin antibiotic within 90 days after DOC, including three who experienced delayed reactions.

## Discussion

Our findings support the use of DOC in practice to remove penicillin allergy labels and optimize antibiotics for patients with low-risk allergies. Despite the high success rate of DOC in our cohort, nearly 18% of patients either did not have their allergy removed from their medical record, or had their allergy added back to their medical record for unclear reasons. Notably, stewardship workflow at our institution involves a best practice alert (BPA) upon allergy re-entry. Upon further chart review, we believe BPAs failed to fire when patients electronically entered penicillin allergies while scheduling initial clinic visits, despite successful PO challenge. Additionally, patients often reported potential alternative causes for delayed reactions, presenting a challenge to discern the nature of the reaction through EHR review alone. These observations may signal a need to focus on patient empowerment and education regarding penicillin allergy de-labeling.

DOC has been increasingly adopted into clinical practice after findings in the PALACE study demonstrated non-inferiority to the historic standard of penicillin skin testing, which requires an abundance of time and resources.^3–4^ The PALACE trial included patients with low-risk penicillin allergies, 95.8% with a PEN-FAST score 0–1, which influenced our institutional recommendations and is reflected in the majority of our cohort having a PEN-FAST score of 0–1 (94.6%).^3^ In the PALACE trial, .5% of participants experienced a positive challenge, defined as an immediate immune-mediated reaction within 1 hour of DOC.^3^ These findings align with our cohort, as one (1.1%) patient experienced an immediate reaction. The PALACE trial also evaluated delayed reactions up to 5 days post oral challenge and reported 22 (11.6%) patients experiencing any delayed adverse effect in the DOC cohort, 9 (4.7%) of which were immune-mediated. The median time to immune-mediated delayed reaction was 4 days.^3^ We collected delayed reactions at any time point after oral challenge and observed a similar rate of 4.3%; however, in the four patients with delayed reactions these occurred a median of 7 days post-DOC. Our cohort study supports the efficacy findings of the PALACE trial and, with longer follow-up than available in current literature, instills confidence that DOC for penicillin allergy removal is safe in low-risk patients for months following the trial period.

Prior to the PALACE study, other institutions published center-specific data supporting the safety and efficacy of DOC for penicillin allergy removal.^5–6^ However, most available data focuses on immediate reaction and allergy removal, and there is a lack of evidence focused on long-term outcomes for these patients. In our review, nearly 18% of patients currently have documented penicillin allergies despite passing DOC without an immediate or a delayed reaction. This indicates a need for systems to ensure a negative DOC is followed by prompt allergy removal, as well as education to avoid re-labeling because of patient or provider uncertainty. Moreover, our data is novel in that we reviewed outcomes of DOC in both the inpatient and ambulatory setting, where most validation data is outpatient-focused.

Our study has limitations. The retrospective nature of our observational study precluded our ability to collect data on definitive reasons for allergy labels being removed or re-entered to the EHR. Additionally, data from a single center may not be generalizable; however, characteristics and findings are consistent with previously published literature.

Our review supports current guidance and published data regarding the safety and efficacy of DOC for allergy removal in patients with low-risk penicillin allergies. It is imperative that institutions take necessary measures to ensure penicillin allergies are removed following successful DOC, and that patients and healthcare team members are informed to avoid unjust allergy re-labeling.

## Supporting information

10.1017/ash.2025.10168.sm001 Harris et al. supplementary material Harris et al. supplementary material

## Supplementary material

The supplementary material for this article can be found at https://doi.org/10.1017/ash.2025.10168.

## Research transparency and reproducibility statement

This study was reviewed and approved by Duke University Health System Institutional Review Board. Informed consent was waived due to the retrospective design. All relevant methods, including patient selection criteria, PEN-FAST scoring, oral challenge protocol, and outcome definitions are described in sufficient detail to allow replication. De-identified data used for this analysis are available from the corresponding author upon reasonable request. No custom code was used for analysis. All data were analyzed using standard descriptive statistics in Microsoft Excel.
