# Supplementary material for: Direct oral amoxicillin challenge in a tertiary care center: validating PEN-FAST in inpatient and outpatient populations with low-risk penicillin allergies
Source: Antimicrob Steward Healthc Epidemiol. 2025 Dec 9;5(1):e333. doi: 10.1017/ash.2025.10168 (PMC12722550; doi:10.1017/ash.2025.10168)
Supplement: Harris et al. supplementary material [file S2732494X2510168Xsup001.docx]

**Supplemental Appendix**

- 1. **Methods**
- Patient follow-up for antibiotic use and delayed reactions was collected via electronic health record review through day 90 after receipt of oral amoxicillin challenge
- PEN-FAST Score Calculations
  - PEN-FAST score was calculated through information collected via electronic health record review. Scores ranged from 0-5. Points were given for anaphylaxis or angioedema (2), < 5 years from reaction (2), and treatment required (1).
  - If patients could not recall if treatment was required, one point would be recorded for treatment unknown
  - If patients could not recall the nature of the reaction, no points were recorded
  1. **Key Definitions**
- Challenge tolerance: Completing direct oral amoxicillin challenge with no documented adverse reaction to testing. Adverse reaction including but not limited to, rash, swelling, hives.
- Iatrogenic immunosuppression: Steroid use > 20 mg prednisone or equivalent for > 2 weeks, chemotherapy for active cancer treatment, tacrolimus, cyclosporine, methotrexate, mycophenolate, azathioprine, everolimus, sirolimus
- Immediate reaction: Within 60 minutes of oral amoxicillin challenge administration
- Delayed reaction: More than 60 minutes of oral amoxicillin challenge administration. Followed for 90 days.
